# Determinants of shisha use among secondary school students in Sudan

**DOI:** 10.1186/s12889-019-7748-3

**Published:** 2019-10-28

**Authors:** Mohammed Othman, Nasrin Aghamohammadi, Nik Daliana Nik Farid

**Affiliations:** 1 Department of Social and Preventive medicine, Faculty of Medicine, University of Malaya, Kuala Lumpur, Malaysia; 2 Centre for Occupational and Environmental Health, Department of social and Preventive Medicine, Faculty of Medicine, University of Malaya, Kuala Lumpur, Malaysia

**Keywords:** Shisha, Smoking, Tobacco, School health, Adolescent, Prevalence, Sudan

## Abstract

**Background:**

Shisha smoking has re-emerged in the Middle East in the last two decades and has spread rapidly in these communities. Information about shisha smoking in adolescents in Sudan is deficient. Hence, the aim of this study is to estimate the prevalence of shisha smoking among adolescents and determine the associated factors.

**Methods:**

This study is a school based cross sectional study among secondary school students in Khartoum State - Sudan that targets both male and female students aged 14–17 years. A total of 3387 students from 29 public and private schools were selected by multi stage random sampling. The participants completed an anonymous self-administered questionnaire which was based on Arabic version of the Global Youth Tobacco Survey (GYTS).

**Results:**

The response rate was 100% in schools and among participants, 57.3% were females and 51.6% were from public schools. The overall prevalence of those who had ever smoked shisha was 13.4%, and among male students the prevalence was 16.8%, while it was 10.9% in females.

The associated factors were poor academic performance OR 2.90 CI 95% (1.21–6.94), friends smoking shisha OR 2.39 CI 95% (1.65–3.45), friends smoking cigarettes OR 2.76 CI 95% (1.90–4.01), peer pressure to smoke shisha OR 13.76 CI 95% (7.86–24.07) and unexpectedly restriction of selling shisha to minors OR 2.21 CI 95% (1.28–3.82).

**Conclusion:**

The prevalence of those who had ever smoked shisha is among the lowest in Middle East region; therefore, regular surveillance system is needed. A well-structured peer based comprehensive tobacco control programmes that are supported by strict and rigorous anti-tobacco regulations which control both commercial and social resources of tobacco are needed to contain this issue among adolescents.

## Background

Shisha smoking is re-emerging as a type of smoking tobacco. For centuries, it was a traditional method of smoking tobacco in Middle Eastern societies. The practice was on the decline in the twentieth century, nonetheless, in the nineties of the last century the practice of shisha smoking saved by the manufacture of flavoured sweetened tobacco [1]. This sweetened tobacco, which is called Maassel, is a fermented type of tobacco with additions of molasses, glycerine, and fruit essences that produce a moist and pliable mixture [2]. Shisha is known by various names such as: waterpipe, hookah, *argilah*, and *nargilah.*

Suddenly, shisha became popular among young people. The quick spread of shisha could be attributed to the revolution of communication at that time such as satellite television and the internet which was used for promotion and advertising for shisha [2].

The social acceptance of shisha as traditional and cultural behaviour enhanced its practice in the family gathering [2]. This acceptance encourages adolescents and young people to smoke shisha with their friends; moreover, the sharing practice of shisha makes it more affordable. Usually, shisha is smoked intermittently in a social context and in sessions that last from half an hour to several hours [2].

Due to the recent re-emerging of shisha, the health effects of shisha smoke are not clear to the public as cigarette smoking. Thus the public perceived that shisha smoking is less harmful and hazardous than cigarettes [3]. Nonetheless, many studies have reported a number of adverse health effects, shisha smoking was associated with oral cancer, oesophageal cancer, gastric carcinoma [4] and lung cancer [5]. Furthermore, carbon monoxide is associated with dizziness and syncope. Acute carbon monoxide poisoning has also been reported with shisha smoking [6]. Coronary heart disease is another major adverse effect of shisha [7]. Moreover, shisha has been associated with a marked decline in pulmonary function values, raising the potential of later suffering from chronic obstructive pulmonary diseases [8]. Additionally, the sharing of shisha mouthpiece is associated with an increased risk of contracting infectious diseases such as tuberculosis [9]. Moreover, dependence is the common health effect of nicotine [10] and also it causes elevated heart rates and increased blood pressure in shisha users [11]. Compared to smoking one cigarette, one session of shisha smoking contains 1.7 times the nicotine, 8.4 times the carbon monoxide, and 36.0 times the tar. Furthermore, it produces a urinary cotinine level that corresponds to smoking 10 cigarettes per day [12].

In Eastern Mediterranean countries, the current use of shisha in adolescents was highest in Lebanon, with 36.9%, followed by Palestine (West Bank) at 32.7%, Syria at 20.1%, and Jordan at 18.9%. The lowest prevalence was reported in Oman with 2.5% followed by Yemen that reported 2.7% of shisha use among adolescents [13].

In Sudan, the prevalence of who had ever smoked cigarettes among adolescents was 21.8% [14] and the prevalence of who had ever used smokeless tobacco was 10.9% [15]. Public Health Act in Sudan prohibited the use of tobacco in schools, universities, hospitals, and worship places, indoor and outdoor public places and in transportation. Moreover, it banned tobacco advertising and promotion and sponsorship in all audio and visual and printed media and it mandate warning messages on tobacco packages. Furthermore, it restrained selling tobacco to minors and the use of shisha was placed under this act. In spite of smoking ban in restaurants and coffee shops in Sudan as in many other countries in the region, the absence of enforced and firm regulation of shisha practice has led to serving shisha in these places [2], moreover, this result in wide availability and accessibility for shisha practice.

Many countries has not addressed the matter of shisha except lately [16]. In case of Sudan, no study to date has addressed the issue of shisha smoking in adolescents except for the Global Youth Tobacco Survey (GYTS) in 2009 that only reported the prevalence of current shisha smoking at 6% [17]. Hence, the aim of this study is to investigate the extent of shisha issue in adolescents in Sudan and to identify its determinants in order to develop and implement policies and regulations to enforce prevention programme.

The determinant factors in this study were categorized in two groups; the first one is the sociodemographic factors which includes; age, gender, grade, locality, location of school, type of school, daily allowance, academic performance, family structure and parental education. The second group includes; exposure to second hand smoke, pro and anti-tobacco messages, parental smoking, friends using tobacco, teachers using tobacco, peer pressure, and restriction of selling shisha to minors. The null hypothesis of the study states that none of the above determinant factors is associated with ever smoking shisha among adolescents.

## Methods

### Study design

This was a school based descriptive cross sectional study targeting both male and female adolescents aged 14–17 years in secondary schools in Khartoum State, Sudan. The state is divided in to three cities that include a total of seven localities. There are two stages of education in Sudan: the primary stage consisting of eight grades followed by the secondary stage consisting of three grades. Khartoum state has about 1000 secondary schools and about 200,000 students [18]. The public schools in the state were 422 schools, of which 216 were boys’ schools and 206 were girls’ schools. While the private schools were 612, among which 357 were girls’ schools and 264 were boys [18] Additional file 1.

The sample size was calculated with following formula $n=\frac{Z^2\ P\ \left(1-P\right)\ }{d^2}$ .

About 3094 students were needed for the study; however, the sample was increased by 10% to overcome nonresponse bias and to aid in the detection of any differences between shisha smokers and non-smokers. At the end 3387 students had participated in the study.

Multistage random sampling was used to recruit the participants for the study. All the schools in the seven localities in the state were stratified by gender and type of school, which resulted in the creation of four strata (male public, male private, female public, and female private). From each stratum, one school was selected through simple random sampling. However, because one female public school lacked the required number of students, another female public school from the same locality was added through the same sampling process. At the end of sampling process, 29 schools were selected to participate in the study, of which 15 were public schools and 14 were male schools. The number of participants needed from each school was determined based on the proportion of students in the locality.

The classes in the selected schools were stratified by grades (first grade, second grade, third grade) and one class was selected from each grade through simple random sampling. All the students in the selected class were invited to participate in the study.

Any male or female student resident in Khartoum State and present in the class at the time of study was included if consent was given. However, any student who was absent at that time was excluded.

### Outcome and predictors

The primary outcome ‘has ever smoked shisha’ was defined as the participants having smoked shisha, even one or two puffs, at any time prior to the study; moreover, if they smoked shisha in the past 30 days they were considered as current shisha smokers. The following sociodemographic characteristics data were obtained from participants: age, gender, grade, academic performance, daily allowance (pocket money), type of school, location of school, locality, family structure, and parental education. The predictors of shisha smoking consisted of exposure to second hand smoke, pro and anti-tobacco messages, parental smoking, friends using tobacco, teachers using tobacco, peer pressure, and restriction of selling shisha to minors.

For the outcome ever having smoked shisha, the question was: Have you ever tried or experimented with shisha smoking, even one or two puffs? Yes/No.

Exposure to second-hand smoke was assessed in five different places: at home, public indoor areas, public outdoor areas, in school, and in transportation.

The issue of parental smoking was addressed by one question: Do your parents smoke cigarettes? The issue of tobacco use by friends was addressed by three questions.

- Do any of your closest friends smoke cigarettes?
- Do any of your closest friends smoke shisha?
- Do any of your closest friends use smokeless tobacco?

Restriction of selling shisha to minors means that the seller should request a proof from the young person that he/she is above the age of 18 years, and if the buyer fails to provide such proof the seller is obligated to refuse to sell this product to the young person. This issue was assessed by one question

- During the past 30 days, did anyone refuse to serve you shisha because of your age?

Lastly, the issue of peer pressure was addressed by one question:

- If one of your best friends offered you shisha, would you smoke it?

The Arabic version of GYTS [19] has been validated by other studies based in the Middle East [20, 21]. Cronbach alpha was 0.79 for smoking habits and it was 0.93 for knowledge and attitude [22]. During the months of September and October 2016, the anonymous self-administrated questionnaire was used to collect the needed data. The questionnaire was pre-tested in 19 students and adjusted accordingly. Those who participated in the pre-test were excluded from the main study. Completion of the questionnaire by participants took approximately 30 to 40 min.

The questionnaire was composed of 76 questions, all of which were close-ended except for two. To ensure that the students answered all the questions, the questionnaire was designed to prevent them from skipping questions.

### Data collection

The study was approved by Medical Ethics Committee University Malaya Medical Center as well as the National Research Ethics Review Committee of the Federal Ministry of Health, Sudan; and permission to conduct the study was obtained from Ministry of Education in Khartoum State. The principals of the selected schools were contacted to obtain their assent of participation after they were provided with the approval forms and the purpose of the study was explained to them. The date of the data collection was set according to the suitability and convenience of schools. Informed written consent was obtained from the participants and parents after they were informed about the aim and objectives of the study and they were informed that participation is optional and voluntary.

Participants were assured that their identity and personal details would not be revealed to any third party and their confidentiality would be secured. Prior commencing the filling of the questionnaire, any inquiries or questions asked by participants were addressed and answered.

### Data analysis

The collected data were analysed using the IBM Statistics Package for the Social Sciences (SPSS) version 20. A complex sample analysis was used because a multistage sampling method was applied in the sampling process; moreover, the weight of each participant was calculated and utilized accordingly. At first, the school weight was calculated in proportion to the number of schools in locality then the class weight was calculated in ratio to the number of classes in school.

The descriptive statistics of sociodemographic characteristics of the participants were reported in frequencies and percentages. The prevalence of smoking shisha was estimated. A general linear model was used to illicit the determinants that may contribute to shisha smoking. A binary logistic regression model was carried out to determine the predictors of shisha smoking. Subsequently, multivariable logistic regression was carried out to develop adjusted model. The adjusted model was created by including the significant sociodemographic characteristics in addition to the significant explanatory variables simultaneously. The *p* value was considered significant if it was less than 0.05 and the result was expressed in odds ratio (OR) with a 95% confidence interval (CI).

## Results

In all, 3387 students participated in the study, which represented a response rate of 100% for both the participating schools and the students alike. Females accounted for 57.3% of students, and 51.6% were from public schools. In all, 78.2% of students were living with both parents, and only 1.1% of students were enrolled at rural schools (Table 1). The mean of daily allowance is 8.8 Sudanese pounds 95% confidence interval (8.57–9.04) and the standard error was 0.119.

**Table 1:** Sociodemographic characteristics of participants

	Weighted Percentage %	Unweighted Counts
Locality		
Khartoum	17.9%	422
Jabal Awliya	14.0%	518
Omdurman	12.5%	459
Karai	11.2%	442
Ombada	17.2%	647
Bahri	10.1%	375
East Nile	17.2%	511
Total	100.0%	3374
Location of school		
Urban	98.9%	3266
Rural	1.1%	108
Total	100.0%	3374
Type of School		
Public	51.6%	2035
Private	48.4%	1339
Total	100.0%	3374
Age		
14 years or less	21.8%	726
15 years	24.8%	879
16 years	28.6%	964
17 years or above	24.8%	783
Total	100.0%	3352
Daily Allowance in Sudanese pounds		
Low <=5	35.7%	1240
Average 6–10	48.8%	1461
Above Average 11–15	9.7%	245
High 16–20	4.7%	107
Very High 21–50	1.0%	19
Total	100.0%	3072
Gender		
Male	42.7%	1604
Female	57.3%	1770
Total	100.0%	3374
Grade		
1st secondary	31.2%	1098
2nd secondary	23.5%	953
3rd secondary	45.3%	1323
Total	100.0%	3374
Academic Performance		
Excellent	20.3%	615
Very good	44.1%	1430
Good	29.7%	1064
Fair	4.6%	160
Poor	1.3%	42
Total	100.0%	3311
Family structure		
Father only	1.8%	74
Mother Only	11.0%	374
Father and Mother	78.2%	2616
Other	9.0%	294
Total	100.0%	3358
Father education		
Illiterate	3.7%	140
Can read and write	12.7%	470
Primary	5.8%	210
Elementary	8.6%	296
Secondary	15.2%	538
University	21.3%	665
Higher Education	12.4%	333
Do not know	20.4%	694
Total	100.0%	3346
Mother education		
Illiterate	8.0%	290
Can read and write	10.4%	375
Primary	9.4%	334
Elementary	11.6%	392
Secondary	19.5%	666
University	19.4%	573
Higher Education	5.9%	173
Do not know	15.9%	543
Total	100.0%	3346

The overall prevalence of those who had ever smoked shisha was 13.4%. Among male students, the prevalence was 16.8%, while it was 10.9% in female participants. The sociodemographic characteristics of those who had ever smoked shisha and who had never smoked were described in (Table 2).

**Table 2:** Sociodemographic characteristics of ever and never shisha smokers

	Never Shisha smoker		Ever Shisha smoker	
	Weighted %	Unweighted Counts	Weighted %	Unweighted Counts
Locality				
Khartoum	85.2%	367	14.8%	53
Jabal Awliya	89.0%	453	11.0%	58
Omdurman	88.4%	407	11.6%	50
Karari	83.3%	368	16.7%	70
Ombada	91.5%	594	8.5%	49
Bahri	82.0%	297	18.0%	72
East Nile	84.8%	417	15.2%	87
Total	86.6%	2903	13.4%	439
Location of school				
Urban	86.7%	2825	13.3%	412
Rural	74.3%	78	25.7%	27
Total	86.6%	2903	13.4%	439
Type of School				
Public	88.7%	1770	11.3%	246
Private	84.3%	1133	15.7%	193
Total	86.6%	2903	13.4%	439
Age				
14 years or less	90.9%	649	9.1%	69
15 years	87.3%	770	12.7%	103
16 years	86.9%	836	13.1%	122
17 years or above	81.6%	628	18.4%	143
Total	86.6%	2883	13.4%	437
Daily Allowance in Sudanese pounds				
Low <=5	87.6%	1070	12.4%	158
Average 6–10	87.3%	1275	12.7%	174
Above Average 11–15	82.7%	201	17.3%	41
High 16–20	83.7%	88	16.3%	19
Very High 21–50	55.9%	11	44.1%	8
Total	86.5%	2645	13.5%	400
Gender				
Male	83.2%	1320	16.8%	269
Female	89.1%	1583	10.9%	170
Total	86.6%	2903	13.4%	439
Grade				
1st secondary	90.8%	983	9.2%	106
2nd secondary	87.6%	829	12.4%	114
3rd secondary	83.2%	1091	16.8%	219
Total	86.6%	2903	13.4%	439
Academic Performance				
Excellent	81.7%	507	18.3%	102
Very good	89.3%	1266	10.7%	152
Good	87.5%	922	12.5%	134
Fair	86.0%	130	14.0%	25
Poor	49.1%	22	50.9%	20
Total	86.5%	2847	13.5%	433
Family structure				
Father only	71.9%	55	28.1%	19
Mother Only	84.2%	312	15.8%	59
Father and Mother	88.1%	2287	11.9%	305
Other	80.0%	237	20.0%	53
Total	86.6%	2891	13.4%	436
Father education				
Illiterate	84.0%	115	16.0%	24
Can read and write	89.3%	412	10.7%	52
Primary	92.6%	194	7.4%	14
Elementary	85.5%	252	14.5%	39
Secondary	86.5%	461	13.5%	72
University	86.2%	575	13.8%	87
Higher Education	81.1%	270	18.9%	59
I do not know	87.8%	599	12.2%	89
Total	86.6%	2878	13.4%	436
Mother education				
Illiterate	85.3%	239	14.7%	44
Can read and write	87.6%	329	12.4%	43
Primary	88.8%	297	11.2%	34
Elementary	88.5%	343	11.5%	45
Secondary	87.5%	583	12.5%	78
University	85.5%	488	14.5%	82
Higher Education	79.3%	134	20.7%	37
I do not know	86.8%	467	13.2%	71
Total	86.6%	2880	13.4%	434

Only locality, daily allowance, gender, grade, academic performance, and family structure were associated with shisha smoking in students. As regards the predictors of shisha smoking, exposure to second hand smoke in transportation, friends smoking cigarettes, friends smoking shisha, peer pressure to smoke shisha, and restriction of selling shisha to minors were the significant predictors for shisha smoking in students. However, when these findings were adjusted for the above-mentioned significant sociodemographic characteristics, only friends smoking cigarettes, friends smoking shisha, peer pressure to smoke shisha and restriction of selling shisha to minors were significant predictors (Table 3). The adjusted model was able to correctly predict smoking shisha in students by 89.2%.

**Table 3:** Crude and adjusted models for independent variables with ever shisha smoking

	Crude Model		Adjusted Model	
	OR CI 95%	*P* value	OR CI 95%	*P* value
City		< 0.001		0.048
Khartoum	1.17 (0.85–1.60)		1.10 (0.77–1.57)	
Omdurman	Ref		Ref	
Bahri	1.83 (1.34–2.49)		1.51 (1.09–2.10)	
Gender		< 0.001		0.754
Male	1.75 (1.33–2.30)		1.06 (0.75–1.49)	
Female	Ref		Ref	
Grade		< 0.001		0.179
1st	0.74 (0.53–1.04)		1.0 (0.68–1.48)	
2nd	Ref		Ref	
3rd	1.53 (1.13–2.08)		1.33 (0.92–1.91)	
Daily allowance	–	< 0.013	–	0.744
Academic performance		< 0.001		< 0.002
Excellent	1.50 (1.05–2.16)		1.23 (0.82–1.84)	
Very Good	0.78 (0.58–1.07)		0.72 (0.50–1.02)	
Good	Ref		Ref	
Fair	0.78 (0.42–1.42)		0.60 (0.31–1.17)	
Poor	5.06 (1.97–13.0)		2.90 (1.21–6.94)	
Family structure		< 0.016		0.147
Father only	2.23 (1.14–4.37)		1.99 (0.96–4.16)	
Mother only	1.5 (1.04–2.17)		1.22 (0.81–1.84)	
Father and mother	Ref		Ref	
Others	1.43 (0.93–2.22)		1.41 (0.87–2.29)	
Parents smoking	–	0.322	–	–
Friends smoking cigarettes		< 0.001		< 0.01
Yes	2.20 (1.58–3.07)		2.76 (1.90–4.01)	
No	Ref		Ref	
Restriction of selling shisha to minors		< 0.002		< 0.010
I did not try to buy shisha	1.36 (0.99–1.87)		1.43 (1.03–1.99)	
Yes	2.49 (1.48–4.20)		2.21 (1.28–3.82)	
No	Ref		Ref	
Peer pressure to smoke Shisha		< 0.001		< 0.01
Yes	11.46 (6.84–19.32)		13.76 (7.86–24.07)	
No	Ref		Ref	
Friends smoking shisha		< 0.001		< 0.01
Yes	2.24 (1.58–3.17)		2.39 (1.65–3.45)	
No	Ref		Ref	

The male gender was a significant predictor for smoking shisha with (OR 1.75 95% CI 1.33–2.30), but it became insignificant after adjustment. Poor academic performance was associated with shisha smoking (OR 5.06 95% CI 1.97–13.0), and it remained significant after adjustment with (OR 2.90 95% CI 1.21–6.94). Friends smoking cigarettes was significant before and after adjustment (OR 2.20 95% CI 1.58–3.07) and (2.76 95% CI 1.90–4.01), respectively.

The strong predictor was peer pressure, which remained strong even after adjustment. Moreover, a similar trend was noticed in the relationship between shisha smoking and friends who smoked shisha.

Surprisingly, restriction of selling shisha to minors was associated with had ever smoked shisha with an (OR 2.49 95% CI 1.48–4.20), and even after adjustment, it continued to be significant (OR 2.21 95% CI 1.28–3.82).

## Discussion

Scant information is available about the prevalence of shisha smoking in Sudan because it is an old traditional method of tobacco smoking in the Middle East and the Indian subcontinent that has only re-emerged in the region during the past two decades. However, it is known that in the last decade it has become more popular in Sudan because it is incorrectly perceived to be less harmful than cigarettes [23].

In Jordan, the percentage of adolescents who had ever smoked shisha is 51.7% [24], and in Lebanon, it is 44.3% [25], while in Saudi Arabia, it is 33.0% [26], and in Egypt, it is 12.6% [27]. Thus, from the above results, it seems that Sudan has the lowest prevalence (13.4%) in the region.

In the Arabian peninsula (Bahrain, Oman, Qatar, United Arab Emirates, Kuwait, and Yemen), boys are significantly more likely to be current shisha smokers than girls except in Qatar [28]. The present study showed that boys were more than girls in being ever shisha smokers; however, the gender difference was not significant. A Lebanese study found that girls are more often to be current shisha smokers, but there is no gender difference, which could be explained by the socioeconomic empowerment of women in Lebanon and the perception that shisha smoking is closer to the local traditions, which means that shisha smoking is not subject to the societal taboos that are associated with smoking cigarettes [29].

In the present study, age and grade of participants showed no significant associations with shisha smoking which is compatible with the results of a study conducted in London, United Kingdom in which it was reported that there is no age difference in those who had ever smoked shisha [30]. However, a Lebanese study found that shisha smoking is significantly associated with age, where those who are older are more likely to have ever and current smoked shisha [25]. In the present study, the age range was too narrow (14–17 years old) to detect any obvious change; the behaviour of students in this age group might not be that different because they study in the same secondary school stage; however the difference may be apparent if a comparison was made between secondary stage and primary stage.

As for the relation between shisha smoking and daily allowance, a Jordanian cohort study among seventh grade students reported a non-significant association between daily allowance and shisha smoking [31], the finding of the present study is consistent with that result. In the case of Sudan, the cheap price of the local brands of shisha as well as the sharing nature of shisha smoking makes it easy for those with lower daily allowance to get shisha. Therefore, it might have contributed to the lack of the association between shisha smoking and daily allowance.

The close resemblance of students in public and private schools and the similar regulations about tobacco use in Khartoum State and within public and private schools might explain the lack of association between type of school and shisha smoking.

Also, the under representation of rural students in this study might be responsible for the lack of difference between urban and rural students, nonetheless, the authenticity of this result cannot be ruled out.

In contrast to many studies [32], the result of the present study showed no association between family structure and shisha smoking. However, the result is similar to an Iranian study that found a lack of association [33]. It seems that shisha smoking is related more to family function than to family structure. The Sudanese community is characterized by extended families, and usually nuclear families are contained and united with in extended families, which provides a more conservative and disciplined environment for adolescents. In the present study, the level of parental education had no significant association with shisha smoking, which was also reported to be the case in other studies [33, 34]. However, many studies have reported that low parental education is associated with smoking in adolescents [35].

This study did find, however, that poor academic performance was associated with shisha smoking. A comparable result was reported in a study among urban high school students in northern Sweden [36]. Problems such as academic difficulties can trigger or reinforce tobacco use in adolescents [37]. On the other hand, this relationship may be inverse, that is to say that tobacco use can cause low academic performance because it may enhance affiliation with peers who discourage academic pursuits [38]. Thus, Tobacco use may contribute to poor academic performance. Alternatively, poor academic performance may lead to the initiation of tobacco use.

Although many studies have confirmed that there is an association between smoking and exposure to second hand smoke at home and in public places [14], the results of the present study did not confirm this association, which means that the findings reported here are similar to those of an Iranian study that showed no association between exposure to second hand smoke and shisha smoking [33].

As regards the selling of tobacco to minors, it has been found that for every 10% increase in sales of tobacco to this group, there is an increase by 0.8% in current smoking in adolescents [39]. However, a longitudinal study reported that tobacco use in adolescents is not affected by reducing the sales of tobacco to minors [40]. The surprising association between restriction of selling shisha to minors and shisha smoking revealed in the present study could be explained by the social availability and access to shisha and the curiosity of adolescents, especially in regards to the recently re-emergence of shisha, even though they are denied access to it. This finding shows that the effect of purchasing tobacco from points of sales is small. Also it seems that adolescents have social resources that can provide them with tobacco even when there are vigorous restrictions of selling tobacco to minors [39]. Thus, reducing tobacco availability can be achieved only when both commercial and social resources are controlled [41].

The present study also found that cigarette smoking in friends was associated with shisha smoking, which is consistent with an Iraqi study among adolescents [42]. Moreover, another cross sectional study in UK reported the same result [16]. Similar to a study in Saudi Arabia [43], the present study revealed an association between shisha use among adolescents and shisha smoking among their friends. Tobacco use by friends is categorized as descriptive norms and the significant association demonstrated here and in previous studies confirms that norms are a valid predictor for tobacco use which is in line with the theory of planned behaviour. The theory proposed that behaviour can be predicted by attitude, norms, and perceived behavioural control [44]. Friends are known to share common attitudes and behaviours, and thus tobacco users may promote pro tobacco attitudes among their friends. Adolescents who use tobacco may select friends with a similar interest in tobacco use; which is known as peer selection. Furthermore, when some friends begin to experiment with or use tobacco, other adolescents may respond by imitating that use [45].

In the present study, when shisha was offered by a friend (peer pressure), the low ability to reject this offer was strongly associated with shisha smoking. This findings is in agreement with that of a longitudinal Jordanian study among students [46]. Inability to reject an offer of shisha may reflect a positive emotional and social perception about shisha use [47], which proves that perceived behavioural control has a tremendous effect on behaviour and hence is a stronger predictor than norms in the model based on the theory of planned behaviour [48].

As regards parental influence, an association between shisha smoking and cigarette smoking by fathers was documented in a large sample size study among Iranian students [33]. However, the present study and another Iranian study found no such association [34]. The difference in the result could be due to the fact that the former study [33] included younger students from elementary and intermediate schools, whereas the later study [34] and the present study recruited participants from high schools only. The inconsistency in the results regarding the relationship between parental smoking and shisha smoking in students could be due to the age of the adolescents. Several studies have investigated this issue. For example, a stronger effect of parental smoking was reported in those aged less than 13 years old, as compared to older adolescents [49]. It has also been found that younger adolescents and children are more affected by parents, whereas older adolescents are more affected by their peers and other role models in society [50]. The above findings may also explain the absence of an association between teachers’ tobacco use and shisha smoking in students in this study as the students’ age was 14–17 years old at the time of the survey.

Lastly, this study found no association between pro tobacco advertising and shisha smoking in students. This is in agreement with another Sudanese study that demonstrated no association between pro tobacco advertising and smoking in adolescents [14]. On the other hand, the present study found that anti-tobacco advertising had no effect on shisha smoking among students, which is similar to findings reported by a study conducted among Croatian students [51]. This result could be attributed to poorly structured, inadequately formulated, and low budget anti-tobacco advertising campaigns in Sudan. Another explanation may be that anti-tobacco advertising might be countering the effect of the pro tobacco advertising, but not yet to the extent of showing decreasing in prevalence [52].

The present study showed that male gender and students with poor academic performance were more frequent to be shisha smokers. Furthermore, cigarette and shisha smoking in friends, peer pressure to smoke shisha and restriction to sell shisha to minors were found to be associated shisha smoking among adolescents in Sudan.

### Strengths and limitations

The main strengths of this study are the large sample size and the high participation rates among the schools and students. However, the under representation of rural students might conceal different characteristics and features in this group who might have different determinants.

Also, the study targeted students only, so the results cannot be generalized to those who have dropped out of school. Furthermore, the inclusion of assessment of shisha smoking among parents and siblings might have added more detail to the findings and led to more comprehensive picture of the phenomenon of shisha smoking among Sudanese adolescent; nonetheless, this was not addressed in this study.

Lastly, it is worth mentioning that no causal relationship can be established from this study as it was a cross sectional study.

## Conclusions

The Assessment of the magnitude of shisha smoking among adolescents represents a useful starting point for a regular surveillance system and the results can be utilized to help develop an effective peer based prevention programmes since the peers have the greatest effect on adolescents. Although the present study found that the prevalence of shisha smoking in Sudan among the lowest in the region, the apparent ineffectiveness of the current anti-tobacco messages should ring alarm bells because this indicates that there is a need for a well-structured and comprehensive tobacco control programmes that should be supported by strict and rigorous anti-tobacco regulations. The association between restrictions of selling shisha to minors with shisha smoking in adolescents demonstrates that controlling tobacco will be achieved only when both commercial and social resources were controlled.

Further research is needed to elaborate more about the shisha issue among rural students and primary stage students; furthermore, to monitor the trend of shisha over the time, also it is essential to address the effect of shisha use in parents and siblings.

## Supplementary information

**Additional file 1.** Sampling Strategy Diagram.

## Data Availability

The data that support the findings of this study are available from Ministries of Health and Education in Sudan. However, restrictions apply to the availability of these data, which were used under license for the current study, and so are not publicly available. Data are however available from the authors upon reasonable request and with permission of Ministries of Health and Education in Sudan.

## Abbreviations

CI: Confidence interval
GYTS: Global Youth Tobacco Survey
OR: Odd ratio
Ref: Reference
