# Supplementary figures and images for: Determinants of shisha use among secondary school students in Sudan
Source: BMC Public Health. 2019 Oct 28;19:1390. doi: 10.1186/s12889-019-7748-3 (PMC6819635; doi:10.1186/s12889-019-7748-3)

**Sampling Strategy Diagram**


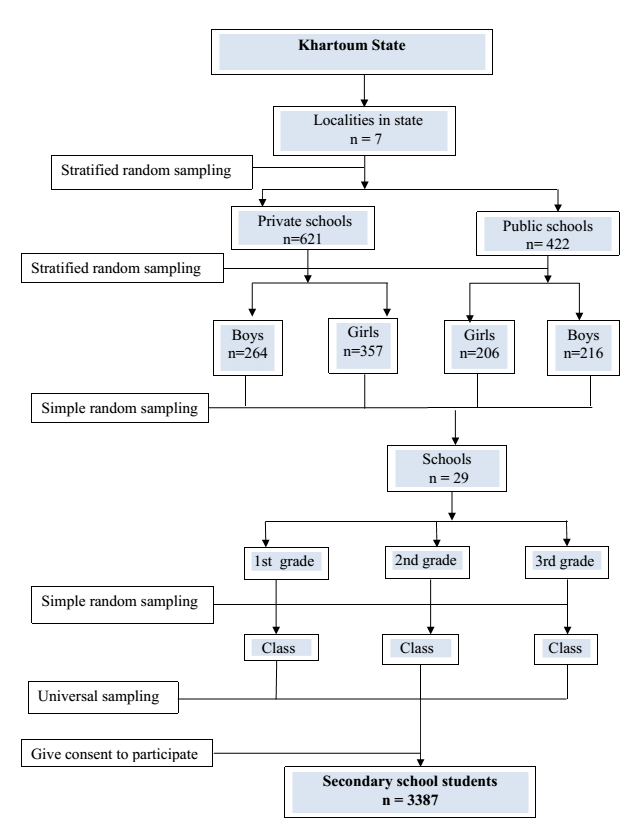

Supplement: Supplementary file 1 — Additional file 1. Sampling Strategy Diagram. [file 12889_2019_7748_MOESM1_ESM.docx]
